# Prevalence of *Mycoplasma bovis* in Algeria and Characterisation of the Isolated Clones

**DOI:** 10.3389/fvets.2022.910799

**Published:** 2022-05-20

**Authors:** Yasmine Oucheriah, Nouzha Heleili, Adélie Colin, Catherine Mottet, Florence Tardy, Claire A. M. Becker

**Affiliations:** ^1^Université de Batna, Laboratoire de Recherche ESPA, Batna, Algeria; ^2^Université de Lyon, Anses, VetAgro Sup, UMR Mycoplasmoses Animales, Lyon, France; ^3^Université de Lyon, VetAgro Sup, Anses, UMR Mycoplasmoses Animales, Marcy l'Etoile, France

**Keywords:** bovine respiratory disease, antimicrobial resistance, Algeria, *Mycoplasma bovis*, genetic diversity

## Abstract

Bovine respiratory disease (BRD) is common in calves in Algeria, but to date, *Mycoplasma bovis* has never been monitored as a potential etiological agent. Here, to assess the presence (direct detection) and circulation (indirect detection) of *M. bovis*, broncho-alveolar lavage fluids (BALF) and serum samples were collected from 60 veal calf farms in Algeria. A commercial ELISA kit (ID Screen^®^ ELISA) was used to screen for the presence of specific antibodies against *M*. bovis in 351 blood sera collected from both diseased and healthy calves, and 69% (241 sera) tested positive. BALFs from the 176 diseased calves were used to screen for *M. bovis* by real-time-PCR (rt-PCR), and 102 (58%) tested positive. A non-exhaustive set of 53 clones were isolated from 44 calves and further subtyped using *polC* gene sequencing. No predominant subtype was found, and two clones exhibited a new subtype. Fourteen clones were further characterized by multilocus sequence typing, and results showed a high degree of genetic diversity, with some clones having new alleles and subtypes. The minimum inhibitory concentrations (MICs) of 5 antimicrobials regularly used to treat BRD was determined on 45 clones. Susceptibility profiles showed very broad diversity, confirming the variety of clones actively circulating. We detected clones with high MICs, including increased MICs of enrofloxacin (*n* = 5). This is the first study to report the presence of *M. bovis* in Algeria in calves with BRD. This research also finds broad genetic and phenotypic diversity in the actively circulating isolates.

## Introduction

Bovine respiratory disease (BRD) is one of the main threats to cattle health, and it leads to significant economic losses in various cattle production systems worldwide (1, 2), especially in the veal calf sector (1). Typical clinical signs of BRD include fever, dyspnoea, coughing, nasal or eye discharge, depression, and anorexia (3). BRD has various causative agents and is strongly associated with poor environmental conditions (low temperature, high humidity, strong drafts, presence of ammonia, etc.) and multiple risk factors including density and other stressors such as transport, comingling, and nutritional disorders (4).

The infectious agents commonly associated with BRD are viruses such as bovine herpesvirus type 1 (BoHV-1), bovine adenovirus (BAdV), bovine viral diarrhea virus (BVDV), bovine coronavirus (BCoV), bovine respiratory syncytial virus (BRSV), and bovine parainfluenza virus (BPiV) (5). These viruses are generally primary obligate pathogens, and most of them facilitate superinfection by bacterial pathogens such as *Pasteurella multocida, Mannheimia haemolytica, Histophilus somni, Trueperella pyogenes*, and *Mycoplasma (M.) bovis* (6). *M. bovis* is an important cause of BRD, particularly the form that manifests as chronic pneumonia (7, 8). The economic impact of *M. bovis* can be severe. In the United Kingdom, it was estimated that *M. bovis* accounted for a quarter to a third of all losses due to respiratory disease (9).

The epidemiology of BRD is well-described in many countries worldwide, but there is very little relevant data available for the African continent, where BRD syndrome is nevertheless endemic (10, 11). In Algeria, BRD in veal calf feedlots has substantial impact on the national economy due to loss of production and cost of treatment.

The aim of this study was to explore the presence of *M. bovis* in veal calves affected by BRD in Algeria, where veal calf farms make up an important part of the local economy. We performed real-time-PCR (rt-PCR) on BALFs in diseased calves and serological assays using ELISA in healthy and diseased calves to capture the presence (direct detection) and circulation (indirect detection) of *M. bovis*. We then characterized the genotypes and the antimicrobial susceptibility profiles of several *M. bovis* clones isolated from rt-PCR positive specimens.

## Materials and Methods

### Sample Collection and Handling

Samples were collected in 2018 and 2019 from different farms undergoing a BRD episode in several “wilayas” (national administrative divisions) of Algeria. Size of the farms ranged from 17 to 450 calves. In each farm, 3 diseased calves with respiratory symptoms such as fever, cough, nasal discharge, lethargy and dyspnoea were selected for BALF collection as described in Le Grand et al. (12). Briefly, after wiping the nostril surface with 70% alcohol, a 100 cm-length 10 mm-diameter catheter (Vygon, France) was inserted medioventrally in the nasal cavity, passed through the larynx and trachea, and gently advanced into the bronchi until it reached a wedge position. Next, 60 mL of sterile 0.9% NaCl was injected into the lungs and immediately aspirated to recover around 30% of the fluid.

For blood sampling, 5 mL of blood was sampled by a jugular vein puncture on 6 animals in each farm: the same 3 animals that showed respiratory symptoms, and 3 apparently healthy animals. Blood tubes were centrifuged at 3,000 rpm for 15 min, serum was collected in each tube and transferred to an 1.5 mL Eppendorf.

All sera and all BALFs were stored at −80°C and transported to the ANSES laboratory on dry ice. As Algeria was in the middle of a foot-and-mouth disease outbreak, all the samples (blood and BALF) were tested by rt-PCR and proved negative (data not shown). Sample processing is summarized in Supplementary Figure 1.

### Serology

Serum samples were analyzed (at 1:40 dilution) using the ID Screen^®^ *Mycoplasma bovis* indirect ELISA kit following the manufacturer's instructions (ID.vet, Grabels, France), as described in Andersson et al. (13). The test was considered valid if the mean value of the positive control was >0.350 and the ratio between the mean positive control and mean negative control was >3. For each serum, the sample-to-positive percentage (S/P %) was calculated using the formula:


$$\begin{array}{l} \frac{S}{P}\%=\left(\frac{OD_{\text{sample}}-OD_{\text{meannegativecontrol}}}{OD_{\text{meanpositivecontrol}}-OD_{\text{meannegativecontrol}}}\right)\times 100 \end{array}$$

The S/P % served to categorize each sample as positive or negative using the cut-off value provided by the manufacturer (positive if S/P % ≥ 50%).

### Nucleic Acid Extraction and Real-Time PCR Detection of *M. bovis* in BALF

Total nucleic acids were extracted from 200 μL of each BALF using a commercial BioExtract^®^ Column kit (BioSellal, France) following the manufacturer's instructions. Extracts were eluted in 50 μL of sterile water, and 1 μL was used to run a commercial TaqMan^®^ real-time PCR kit for *M. bovis* detection (LSI VetMAX™ *M. bovis*, Life Technologies, France). As suggested before (14), only results with a cycle threshold (Ct) <37 were considered positive.

### Selection of Clones

Once the BALF samples were defrosted for DNA extraction, a 200 μL aliquot was inoculated in 1,800 μL modified PPLO broth as previously described (15). The cultures were incubated at 37°C with 5% CO_2_ for 3 to 4 days.

At the beginning of the study, all samples were cultured in broth and then agar. After the first 10 farms, only samples that tested rt-PCR-positive for *M. bovis* and showed bacterial growth-related turbidity in broth were submitted to further analysis. The BALF subcultures were serially diluted 10-fold in liquid medium to 10^−1^-10^−4^ and incubated for 2 to 3 days. For each sample, the lowest positive dilution showing turbidity was filtered through a 0.22-μm membrane filter, and 10 μL of this filtered broth was seeded on PPLO agar plates added with 0.1% Tween 80 to prevent growth of *M. bovirhinis* (16), then incubated at 37°C with 5% CO_2_ for 48 h. *M. bovirhinis* is a commensal organism of the bovine airways that can hamper the isolation of *M. bovis* as it grows faster. A maximum of 3 colonies per sample were randomly selected then picked with a wooden toothpick and further cultured in 4 mL PPLO broth. Each isolated colony-forming unit (cfu) was then considered a clone. Clones were identified using membrane filtration dot-immunobinding tests (MF-Dot) as described by Poumarat et al. (17).

### PCR and Sequencing for Subtyping Clones

Genomic DNA of each *M. bovis* clone was extracted from 200 μL of culture using a QIAamp^®^ DNA Minikit (Qiagen, Germany) following the manufacturer's instructions. All clones were subtyped using *polC* sequence analysis as previously described (18). A panel of 14 clones was selected (based on geographical origin (wilayas) and *polC* subtype) and analyzed by MLST. The loci *dnaA, gltX, gpsA, gyrB, pta-2, tdk*, and *tkt* loci were amplified by PCR (19, 20). Sanger sequencing of PCR products was outsourced (Genewiz, Germany or Genoscreen, France). The sequences obtained were analyzed using Geneious software (BioMatters, New Zealand). Subtypes were attributed using the PubMLST database for *M. bovis* (https://pubmlst.org/bigsdb?db=pubmlst_mbovis_seqdef&page=profiles). New alleles and subtypes were defined and deposited at https://pubmlst.org/bigsdb?db=pubmlst_mbovis_seqdef. The newly-defined subtypes were compared to the closest analogs found in PubMLST. A BURST analysis was conducted using PHYLOViZ (https://online.phyloviz.net/index), with one clone/isolate per country and per subtype.

### Antimicrobial Susceptibility of *M. bovis* Clones

MICs of 5 molecules (i.e., oxytetracycline, tylosin, florfenicol, enrofloxacin and spectinomycin) corresponding to the antimicrobial families most frequently used in Algeria to treat BRD were estimated on a panel of clones, using the agar dilution method (21). Only two concentrations of each antimicrobial were tested, i.e., the CLSI-standard clinical breakpoints for susceptibility and resistance for *Pasteurellaceae*, a family of bacteria that are known to colonize the same body niche in cattle (22). These concentrations were 2 and 8 μg/mL for oxytetracycline, 32 and 128 μg/mL for spectinomycin, 2 and 8 μg/mL for florfenicol, 0.25 and 2 μg/mL for enrofloxacin, and 8 and 64 μg/mL for tylosin (using the threshold for tilmicosin). Each MIC assay was run twice, and results were never divergent between experiments.

## Results

### Description of the Farms Sample

In total, we sampled 351 animals [age: 3 days−22 months] from 60 farms. Average size of the farms visited was 60 [17–450] head of cattle. Four categories of farms were visited: 45 calf rearing farms (75%), 7 mixed farms (12%) (dairy operations combined with calf rearing units), 6 pilot calf rearing farms (10%), and 2 pilot mixed farms (3%). Algerian farms are mainly made up of dairy cows: calves that are not kept for renewal (males mainly) are either raised on-farm (mixed farms) or quickly sold off to calf rearing farms that grow out the calves for a short period (6 months). Calves are fed with mother's milk or powdered milk. Average age of calves at weaning is 2 months. Cleaning and disinfection of the premises was frequent (cleaning 1 to 3 times a day, disinfection twice a year) in the majority of farms visited (81%, *n* = 49). For parasite control, 38% of farms used animal treatments, with ivermectin being the most common. The majority of calves (90%) were vaccinated against foot-and-mouth disease, as the study was led in the midst of a pandemic. All farms were brucellosis-free and tuberculosis-free. The pilot farms, which receive financial support from the Algerian government, are more modern operations that employ trained staff, including a permanent veterinarian dedicated to herd health management. In these establishments, the owners are fully committed to good hygiene and controlled nutrition practices.

### Detection of Antibodies Directed Against *M. bovis* by ELISA

Individual prevalence of *M. bovis* antibodies was 69% (*n* = 241/351 calves), with 31% sera (*n* = 110) tested negative by ELISA. Out of all 60 farms, 88% (*n* = 53) presented at least one *M. bovis*-seropositive animal, proving a high seroprevalence of *M. bovis* in Algeria.

In all, 134 (76%) of the 176 asymptomatic healthy calves were seropositive, whereas only 107 (61%) of the 175 calves showing various respiratory symptoms were seropositive.

Proportion of seropositive animals was higher in calf rearing farms than in mixed farms (72% vs. 46%) and much higher in traditional farms than in pilot farms (72% vs. 41%) (Table 1).

**Table 1:** Proportions of seropositive animals in each type of farm.

	**Traditional farms**	**Pilot farms**	**Total**
Calf rearing farms	76% (204/269)	44% (16/36)	72% (220/305)
Mixed farms	49% (18/37)	33% (3/9)	46% (21/46)
Total	72% (222/306)	41% (19/45)	69% (241/351)

*Numbers of animals are indicated in brackets*.

### Detection of *M. bovis* by Real-Time PCR

BALFs were collected from 176 diseased calves from all 60 farms (3 calves per farm except for 6 farms). Rt-PCR detected *M. bovis* in 102 out of 175 BALFs (58%) with a Ct ranging from 16.1 to 36.9. The proportion of rt-PCR positive animals was of 62% (66/107) and 53% (36/68) in seropositive and seronegative animals, respectively.

**Figure 1:**
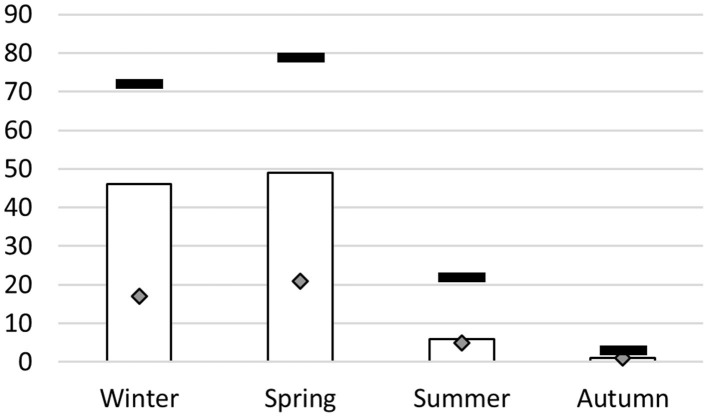
*M. bovis* detection by real-time PCR according to season of sampling. X-axis, season of BALF collection; y-axis, number of calves that tested positive in real-time PCR (target *M. bovis*) (white bars), total number of calves sampled for each season (black dashes) and number of calves from which clones were isolated (diamonds).

Samples were realized in different seasons (Figure 1). Among the 72 calves sampled in winter, 46 (64%) were positive by rt-PCR. Practically the same proportion was observed for calves sampled in spring (62%, i.e., 49 calves out of 97). Numbers of diseased and thus sampled calves were much lower in summer and autumn (Figure 1), so we did not calculate and compare summer and autumn prevalence rates against the other seasons.

### Selection and Genetic Characterization of *M. bovis* Clones

In order to isolate *M. bovis* clones, 174 BALFs were cultured in broth while waiting for the rt-PCR results: 86 cultures (48%) showed characteristic turbidity of the broth while 17 others had an ambiguous turbidity, both indicating potential growth of *Mycoplasma* spp. In total, 116 broths (corresponding mainly to samples positive by both rt-PCR and culture, *n* = 75) were seeded on agar plates. Only half of the rt-PCR-negative broths were tested (*n* = 34 out of 73), and none enabled to isolate any clone. In total, we isolated 156 clones with *Mycoplasma* spp.-compatible morphology from 52 plates. Clones were identified using the MF-dot technique (17). Once one clone per calf was identified as *M. bovis*, other clones were not analyzed further. A final total of 53 *M. bovis* clones were obtained from 43 calves coming from 26 farms (see details in Supplementary Table 1).

The 53 *M. bovis* clones were subtyped using the *polC* sequence (Supplementary Table 1). We found no one predominant subtype: 42% (*n* = 22/53) were st1, 23% (12/53) were st2 and 32% (17/53) were st3. Two clones had an undetermined subtype that differed from st2 by only one SNP.

**Figure 2:**
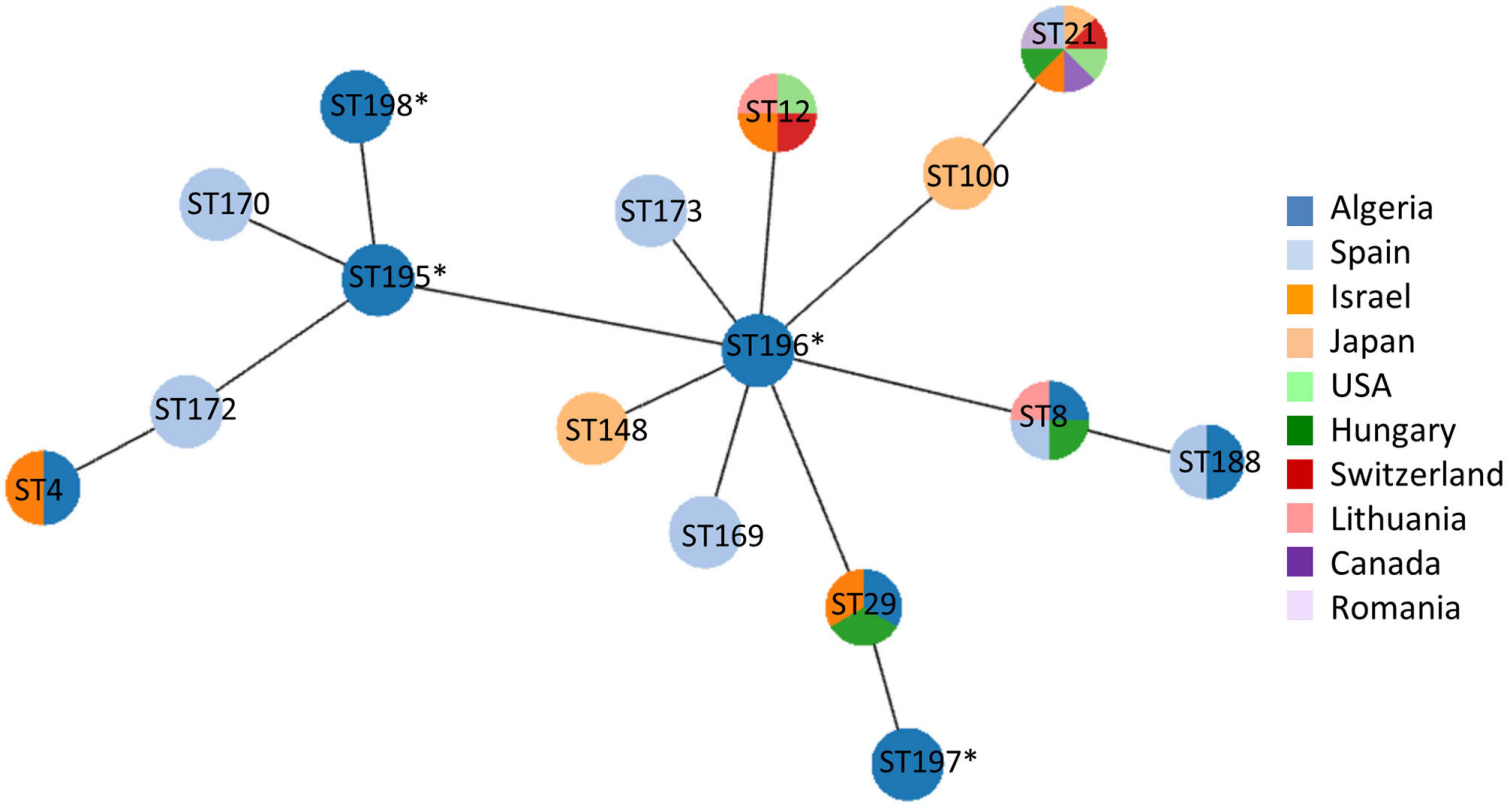
eBURST analysis of the allelic profiles of the Algerian clones (ST4, ST8, ST29, ST188, ST195-198) compared to the closest subtypes found in the PubMLST database. Subtype numbers retrieved from the PubMLST database are indicated in circles, and the new subtypes reported in this study are tagged with an asterisk. Only subtypes close to or common to Algerian isolates are indicated, and their country of origin is color-coded: dark blue, Algeria; light blue, Spain; orange, Israel; light orange, Japan; light green, USA; green, Hungary; red, Switzerland; pink, Lithuania; purple, Canada; lilac, Romania.

A selected panel of 14 clones encompassing phylogenetic and geographic diversity (at least one clone in each wilaya per *polC* subtype) were then characterized by MLST (19, 20). Nine out of these 14 clones were of previously described subtypes (ST4, 8, 29, 188) whereas the other five presented either new alleles (allele 34 for *gyrB* or allele 32 for *pta2*) or new combinations of known alleles resulting in the new subtypes 195–198 (Supplementary Table 1). Those new combinations are close, i.e., differing by only one locus, to subtypes that have recently been added in the database, namely ST100, ST148, ST169, ST170, ST172, and ST173. e-BURST analysis of the Algerian clones and these subtypes was done with PHYLOViZ including the country of origin (Figure 2) and showed that Algerian clones shared some subtypes with isolates sourced from Israel (ST4 and ST29) or European countries (ST8 and ST29, from Lithuania, Spain or Hungary). The other Algerian subtypes were close to subtypes detected from all over the world.

### MIC Evaluation of *M. bovis* Clones in Algeria

**Figure 3:**
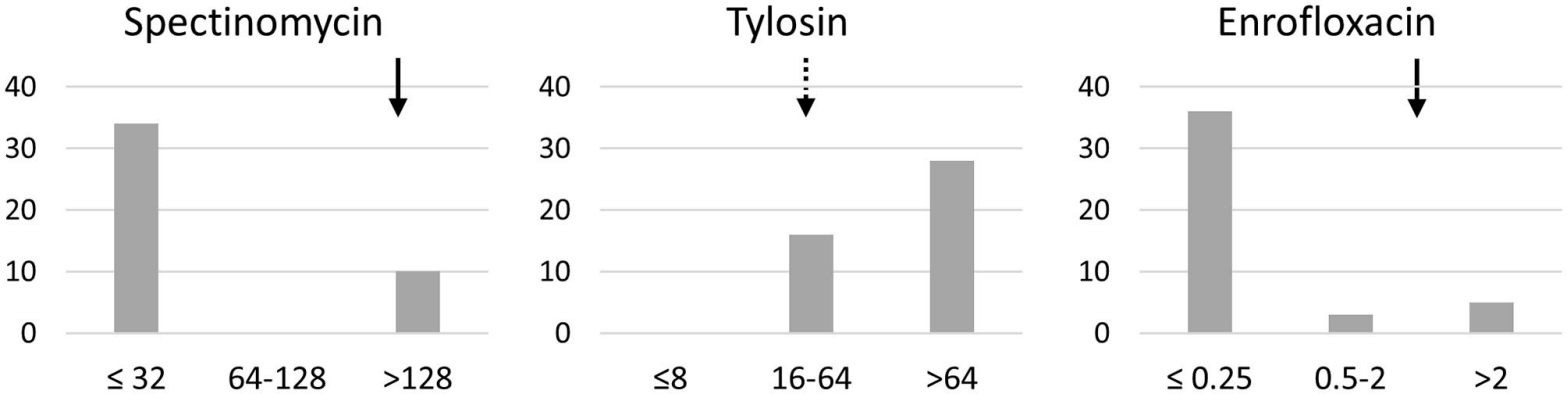
Distribution of the MIC profiles of spectinomycin, tylosin and enrofloxacin for 44 clones of *M. bovis*. X-axis, MICs classes; y-axis, number of clones. Arrows indicate the clinical breakpoints for resistance for *Pasteurellaceae*, i.e., spectinomycin ≥128 μg/mL, enrofloxacin ≥2 μg/mL, and tylosin ≥32 μg/mL threshold of tilmicosin used (dashed arrow).

The MIC of 5 antimicrobials were determined on a panel of 45 clones. One clone per calf was chosen, except for one calf with different subtypes between clones. One clone failed to culture despite numerous attempts. All clones were oxytetracycline-resistant with MIC >8 μg/mL. For florfenicol, all clones showed 2 < MIC ≤ 8 μg/mL, i.e., intermediate or resistant. Results for spectinomycin, tylosin and enrofloxacin are presented in Figure 3. The majority of clones (34/44) were susceptible to spectinomycin whereas they were mainly intermediate or resistant to tylosin. The majority of clones were susceptible to enrofloxacin (36/44), but a not-negligible proportion (5/44) were enrofloxacin-resistant.

## Discussion

To the best of our knowledge, this is the first report of *M. bovis* seroprevalence in Algeria. Out of 351 serum samples, 69% were *M. bovis*-positive, regardless of whether the calves sampled were diseased or not. This prevalence is almost as high as in other reports worldwide studying populations in feedlots with numerous risk factors for BRD. For example, in Italy, 76% of beef cattle and 100% of veal calves presenting pneumonia at slaughter were found to be carriers of antibodies against *M. bovis* (23). Similarly, veal operations in France and Belgium also tested 100% or close to 100%-positive for *M. bovis* antibodies (24, 25). Few studies have addressed *M. bovis* on the African continent, as the main concern in terms of mycoplasmosis is *M. mycoides*, the etiological agent of contagious bovine pleuropneumonia, which is a notifiable disease for the OIE (26). In Nigeria, two studies reported an *M. bovis* seroprevalence of 66% and 19.5% of animals when testing semi-extensively or extensively-managed cattle (27, 28). The surprisingly low figure (19.5%) was attributed by the authors to potentially inadequate sample storage (28).

In our population, we found a slightly higher proportion of healthy-but-seropositive calves than diseased-and-seropositive calves. The kit used here is known to be highly sensitive and thus to detect any contact with *M. bovis* or circulation in a herd (seroconversion after exposure) rather than indicating true clinical infection of the animal (29). This means that the serological results of the calves studied here may not be correlated with clinical status but with time of sampling after onset of on-farm infection or comingling. Given the relatively long time needed for antibodies to appear [9 to 21 days after infection (30)], diseased animals might not yet have all been seropositive at the time of sampling. A greater proportion of animals aged > 1 year old were seropositive (data not shown), showing that the older they were, the more likely they were to have been in contact with *M. bovis*. These results should thus be interpreted with caution, as healthy and diseased animals came from the same farms and were thus exposed to the same conditions.

BRD is known to have multiple zootechnical risk factors that will influence disease outbreak or contamination, including animal housing management with general hygiene and atmosphere of the building, number of animals, and age of individuals. Here, *M. bovis* seroprevalence was higher in calf rearing farms than in mixed farms (72% vs. 46%). This might be associated to comingling of animals from various origins, which is a known significant risk factor for *M. bovis* introduction and spread (31). There was also a lower seroprevalence in pilot farms than in traditional farms. Better allocation of resources (finances and competent staff) to bovine health management could contribute to reduce *M. bovis* spread in the farm.

In Algeria, there have been very few monitoring studies on *Mycoplasma spp*. Two studies have been conducted on contagious agalactia (32, 33) in small ruminants, but neither addressed *M. bovis*. The rt-PCR used here to detect *M. bovis* in BALF samples found a relatively high prevalence (58%). A few samples (*n* = 26, data not shown) were screened by rt-PCR to detect various pathogens using a commercial screening kit and showed co-detection of the usual *H. somni, P. multocida, M. haemolytica*, BCoV, RSV, and BVDV (5, 34, 35). More samples were collected during winter and spring than summer and autumn, as veterinarians reported more diseased calves, which is consistent with environmental conditions favoring *M. bovis*-associated diseases and BRD in winter and spring [Figure 1 (7, 36)]. In a context of BRD in feedlots, our prevalence results for *M. bovis* were similar to other studies, i.e., 51–60% of calves in France (25, 35), or 68% for *Mycoplasma spp*. in Brazil (37). A small sample in Spain (*n* = 23) showed a higher prevalence of *M. bovis* (86.9%) (38) but this may not be representative of the prevalence across the country. We can thus conclude that the epidemiology of *M. bovis* in BRD in Algeria is the same as everywhere else in the world. The sole concern was the predictive value of our method to determine the seropositivity of a farm. The same number of animals was sampled in each farm, whatever its size. This may have induced a bias, so we did not interpret results at the farm level.

The rt-PCR was also used as a strong indicator of potential success of *M. bovis* isolation in cultures. Out of the 43 calves that gave samples enabling isolation of clones, all were rt-PCR-positive, and 42 were also positive for *Mycoplasma* spp. in broth culture (visible turbidity in PPLO-like broth) with the last being doubtful. However, in some cases (38 out of the 81 rt-PCR positive tested for clone isolation), it was impossible to isolate *M. bovis* in culture, even in BALFs with low Ct. Mycoplasmas may have been present but not viable any more or hampered by other species. Some of the MF-dot-screened clones (*n* = 24) were shown to belong to other *Mycoplasma* species, including *M. arginini* (7/24), *M. canadense* (1/24) and *M. alkalescens* (3/24), and 7 calves presented mixed infection with *M. bovis*. This is the first report of these species in Algeria. The culture method used here prevented the growth of *M. bovirhinis* on agar plates (16), so this species was only isolated once among the identified clones. Further studies are needed to complete the full picture of the prevalence of *Mycoplasma* spp. in Algeria.

The subtypes found in Algeria were compared against French currently circulating subtypes, as determined using *polC* sequencing (18). Several differences were noticed regarding the relative proportion of st1, st2, and st3. The st1 subtype was the most frequent in our Algerian population, whereas it has been described as receding in France (18) and is not present in Spain (39). The MLST scheme described by Register et al. (19, 20) was used here to enable a more discriminatory and larger comparison at a worldwide scale. Of the 14 clones, 8 different subtypes were identified, of which 4 were new, which proves the broad diversity of the clones circulating in Algeria. One subtype, ST29, was encountered for 4 clones belonging to two different *polC* subtypes (st1 and st3), which was possible as different housekeeping genes were targeted (Supplementary Table 1). The high plasticity of the *M. bovis* genome, with mobile genetic elements and mycoplasmal chromosomal transfer, may also have contributed to the observed diversity (38). However, whether or not these events are frequently encountered in the field remains to be determined.

We compared the diversity of clones in this study against the distribution of similar subtypes around the world. Unfortunately, with the recent change in MLST scheme (19, 20) the scientific community has lost a large amount of valuable data on *M. bovis* isolates. Many isolates deposited in the legacy scheme have not been re-subtyped. Moreover, new isolates corresponding to already described subtypes are reported in the literature but not submitted to the pubMLST data basis. We completed the PubMLST database, as no data were available for Algeria. We defined new alleles and new subtypes for Algerian clones, which illustrates the genetic diversity in this country, but 8 clones had subtypes that had already been encountered elsewhere, i.e., ST8, ST29, ST4 [described in Israel (40)] and ST188 (described in Spain, PubMLST database). ST8 could be considered as a European one, as it has been described in Hungary and Lithuania (40) and in Spain (39) and is the dominant subtype encountered in France (41). In the database, ST29 only contains isolates from Israel and one from Hungary (40), whereas it has been largely reported in Europe, including Cyprus (42), Denmark, Finland, the Netherlands and Sweden (41). The other closest subtypes (S12, ST21, and ST100) have also been found in these Nordic European countries (adding Estonia) and France (41). The overlap of these Algerian clones with subtypes distributed worldwide proves once again that the cattle trade has to be leading the epidemiology of *M. bovis* (40, 43). Cattle production in Algeria is highly dependant on international trade, and cattle are imported predominantly from France and Spain data available at https://oec.world/en/visualize/tree_map/hs92/import/dza/all/10102/2019 (44). The genetic diversity in Algeria may be similar to that in Spain, with common subtypes observed between these two countries (namely ST8 and ST188), but Algeria also counts several other subtypes that are also present elsewhere or newly observed. It is not possible to determine whether these subtypes have been endemic in the country for a long time and have since evolved locally due to different selection pressures. Alternatively, there may be regular introductions of new subtypes, resulting in important diversity, as in Israel (40). We could also posit that the *polC* st1 had been introduced from France, where it was largely present in the past (18), and has since stayed and evolved in Algeria (as it now belongs to three different MLST subtypes, namely ST4, ST29, and ST198) unlike in France where it has receded. A broad whole-genome sequencing approach would help decipher further the evolution of Algerian isolates.

Another objective of this study was to assess the level of antimicrobial resistance in Algeria in relation to antimicrobial use. Forty-four *M. bovis* clones were tested against 5 antimicrobials covering the antibiotics most frequently used to control respiratory diseases in Algeria. All clones were oxytetracycline-resistant, as described all over the world (45–47). Compared with the resistance levels of European strains (21, 48), Algerian clones showed similar results for spectinomycin and florfenicol. The clones belonging to ST8 showed similar levels of resistance to isolates from France, from where ST8 might have been imported. The proportion of fluoroquinolone-resistant clones in Algeria is intermediate between France and Spain, where the use of fluoroquinolones is, respectively, restricted or not (21, 39). This is consistent with the one enrofloxacin-resistant Algerian clone that was suptyped and belongs to ST188, also present in Spain.

A few calves from which clones were isolated (*n* = 6) had been treated with antimicrobials before sampling (data not shown). Once again, we were unable to find a link between treatments and antimicrobial resistance (35). However, the use of antimicrobials might have jeopardize our capacity to isolate *M. bovis* in a few diseased calves. Out of the 25 treated calves, 19 tested rt-PCR-positive (mean Ct = 29.5, [16.1–36.8]) but *M. bovis* had been isolated in only 5 of them.

The clones found here were proven resistant to at least one molecule (oxytetracycline) but could also be resistant to various combinations of one, two or three additional molecules (spectinomycin, tylosin and/or enrofloxacin) (Supplementary Table 1). This phenotypic diversity contrasts with the situation in France (18, 49), which again points to regular cattle-importation from different origins. It is always tempting to make the connection between molecular subtype diversity and resistance profiles, but there is still no typing method strictly correlated to resistance phenotypes for *M. bovis* (43). Here we confirmed a non-reciprocal link between subtypes and susceptibility profiles: all the clones with increased MIC to enrofloxacin that were resistant to the 4 other molecules belonged to st2 (*polC* typing) (as in French situation) or ST8 and ST188 (MLST typing), but not all the st2 clones were resistant to all molecules tested.

## Conclusion

This study demonstrated for the first time that *M. bovis* is highly prevalent in BRD cases in Algeria. Results on pilot farms in Algeria seem to suggest that improving skills in bovine health management is a vital pathway to limit the economic impact of BRD in Algeria. The clones studied, which were isolated from different farms and wilayas, showed varied subtype profiles and antimicrobial resistance levels. The MLST scheme used here proved a convenient and powerful tool for investigating the epidemiology of *M. bovis* and its links to global cattle trade. We anticipate this study as a cue to prompt more scientists to complete the database so that the community can get a better vision of *M. bovis* around the world. After this first approach, the epidemiology of *M. bovis* in Algeria may actually gain from more in-depth phylogenetic studies to retrace the history of *M. bovis*.

## Data Availability Statement

The original contributions presented in the study are included in the article/Supplementary Material, further inquiries can be directed to the corresponding author.

## Ethics Statement

Ethical review and approval was not required for the animal study because only veterinary clinical samples were done for health monitoring of diseased animals.

## Author Contributions

YO, CAMB, FT, and NH contributed to conception and design of the study. YO did all the cattle sampling. AC, CM, YO, and CAMB performed the laboratory analyses. YO, CAMB, and FT wrote the first draft of the manuscript. All authors contributed to manuscript revision, read, and approved the submitted version.

## Funding

YO received a partial grant from the University of Batna to help cover her stays in France.

## Conflict of Interest

The authors declare that the research was conducted in the absence of any commercial or financial relationships that could be construed as a potential conflict of interest.

## Publisher's Note

All claims expressed in this article are solely those of the authors and do not necessarily represent those of their affiliated organizations, or those of the publisher, the editors and the reviewers. Any product that may be evaluated in this article, or claim that may be made by its manufacturer, is not guaranteed or endorsed by the publisher.
