# Supplementary figures and images for: Prevalence of Mycoplasma bovis in Algeria and Characterisation of the Isolated Clones
Source: Front Vet Sci. 2022 May 20;9:910799. doi: 10.3389/fvets.2022.910799 (PMC9163989; doi:10.3389/fvets.2022.910799)

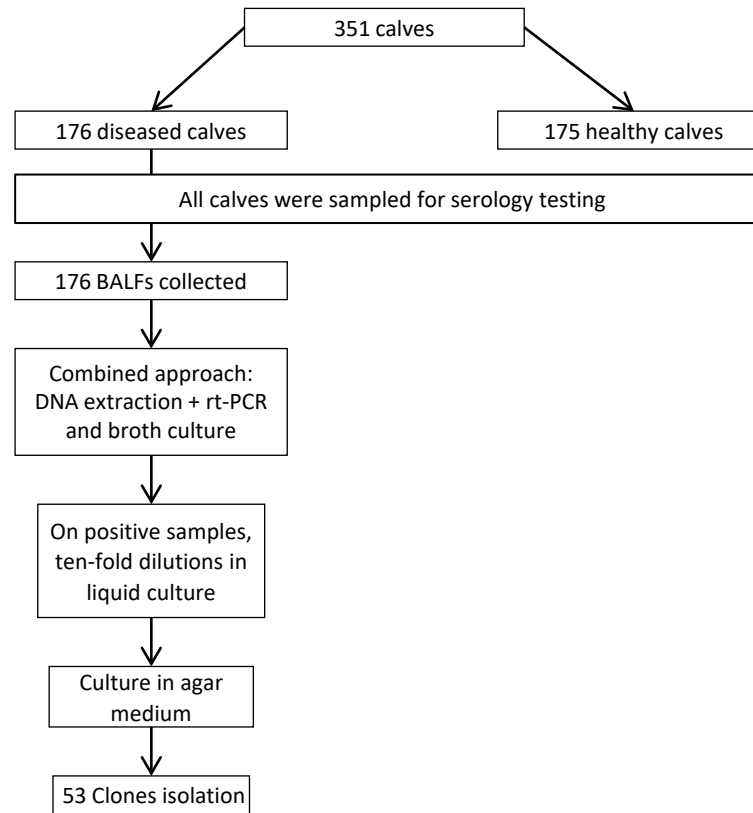

Supplementary Figure 1. Samples processing

Supplement: Supplementary file 2 [file Image_1.pdf]
